# Dietary Patterns and Nutritional Status in Bariatric Surgery Candidates—A Cross-Sectional Study

**DOI:** 10.3390/nu17040716

**Published:** 2025-02-18

**Authors:** Kamila Sobas, Edyta Suliga, Piotr Bryk, Stanislaw Gluszek

**Affiliations:** 1 Institute of Health Sciences, Collegium Medicum, Jan Kochanowski University, 25-369 Kielce, Poland; edyta.suliga@ujk.edu.pl; 2 Dietary Clinic Meduniv sp. zo.o. in Kielce, 25-369 Kielce, Poland; 3 Institute of Medical Sciences, Collegium Medicum, Jan Kochanowski University, 25-369 Kielce, Poland; piotr.bryk@ujk.edu.pl; 4 Clinic of General, Oncological and Endocrinological Surgery, Provincial Hospital in Kielce, 25-736 Kielce, Poland; 5 Department of Surgical Oncology, Holy Cross Cancer Centre in Kielce, 25-734 Kielce, Poland; sgluszek@wp.pl; 6 Department of Surgery, Hospital of the Ministry of Interior and Administration in Kielce, 25-375 Kielce, Poland; 7 Institute of Genetics and Animal Biotechnology, Polish Academy of Sciences, 05-552 Magdalenka, Poland

**Keywords:** degree of obesity, dietary patterns, bariatric surgery

## Abstract

Background/Objectives: Observing a patient’s dietary behaviour before bariatric surgery may help to predict their diet (and indirectly, the rate of weight loss) after the procedure. Consequently, the aim of this study was to identify dietary patterns (DPs) in bariatric surgery candidates, as well as to assess the relationship between DPs, degree of obesity, and body composition. Methods: The participants were comprised of 117 bariatric surgery candidates. Data concerning their diet, lifestyle, and socioeconomic status was collected using the KomPAN^®^ questionnaire. The following three DPs were identified using a principal component analysis: ‘Sandwiches & Sweets’, ‘Fast Food, Convenience Food & Alcohol’ and ‘Prudent’. Baseline nutritional status and body composition using electric bioimpedance were assessed. Results: Differences were found between the DPs, degree of obesity, and body composition. The Prudent DP primarily involved a high consumption of healthy products. Following the Prudent DP, differentiated the degree of obesity and the patient’s body composition the most. In turn, the Fast Food, Convenience Food & Alcohol DP was associated with a higher likelihood of Degree III obesity and a very high visceral fat level (VFL). The Sandwiches & Sweets DP included the most participants with a very high VFL. Conclusions: The bariatric surgery candidates were shown to follow different diets, and different DPs could be identified. Patients with a higher degree of obesity followed a more beneficial DP, which was likely due to their higher awareness of the risks of morbidity in obesity and of post-surgical complications. Socioeconomic factors may attenuate the association between diet and the degree of obesity and body composition in bariatric surgery candidates.

## 1. Introduction

Obesity is a multifactorial disease. Various biological, social, and economic factors influence the probability of obesity. In developed countries, obesity is prevalent among people facing challenging socioeconomic conditions. It may stem from restricted access to healthcare and nutritious food, along with increased stress and anxiety levels [1,2]. All of the above might lead to a higher intake of energy-rich foods and, as a result, to unhealthy weight gain.

Bariatric surgery (BS) is currently the most effective form of treatment used to reduce body mass and decrease mortality in patients with severe obesity. On the other hand, BS may introduce new clinical problems, complications, and side effects, especially problems related to nutrition. Consequently, the diets of bariatric patients require particularly careful planning. Once a patient has qualified for BS, they should be given comprehensive care to prepare them for the treatment [3]. The process should emphasise dietary education, including an assessment of the current diet, identification and correction of any dietary errors, and consultations concerning the patient’s previous attempts at losing weight and the factors that contribute to their weight gain [4,5]. A plan should be designed in order to correct the patient’s current diet and lifestyle [6,7], and should lead to a loss of weight before the BS. Researchers have indicated that losing weight in the pre-surgical period reduces the risk of post-surgical complications, blood loss during the procedure, and the duration of the procedure, compared to patients who have not lost weight [8,9].

The results of some studies suggest that although the total food energy in bariatric patients decreases after the procedure, their dietary preferences do not change [10,11]. This decrease in food energy in the diets of bariatric patients does not result from a change in their preferences towards foods with lower energy density, but rather from eating smaller portions of the same foods as before the procedure. Additionally, the diets of bariatric surgery candidates are not uniform. This means that observing a patient’s dietary behaviour even before the procedure may help to predict their diet (and indirectly, the rate of weight loss) after the procedure and could help to identify patients who will need special support after the procedure (food education) due to their harmful preferences and eating habits [11,12]. Consequently, the aim of this study is to identify the dietary patterns present in bariatric surgery candidates and to assess the relationship between DPs and the degree of obesity and body composition.

## 2. Materials and Methods

### 2.1. Data and Sample Collection

The study was conducted between January 2019 and November 2023, with 125 bariatric surgery candidates. The participants were enrolled among the patients at the Department of General, Oncological and Endocrine Surgery of the Provincial Polyclinic Hospital in Kielce, Poland. The inclusion criterion was a diagnosis of obesity, confirmed by a surgeon, according to the guidelines of the Polish Society for the Treatment of Obesity from May 2022 [6]. The exclusion criteria were an age below 18 or above 65 years, intestinal inflammation, mental disorders, pregnancy and lactation in women, and persons with an electrical device implanted in their heart. The material was collected from among patients referred to the Meduniv sp. z o.o. Dietary Clinic. The research was conducted with the approval of the Bioethics Committee at the Collegium Medicum of Jan Kochanowski University in Kielce, granted on 26 March 2018 (Approval Number: 24/2018). The gathered data were validated and purified by eliminating respondents with incomplete personal details and inconsistent information submitted in a survey. The final baseline data set included 117 patients with obesity. Data regarding the participants’ diet, lifestyle, and socioeconomic status become amassed through the use of the KomPAN^®^ questionnaire [13]. This validated multi-factor food frequency questionnaire was designed for a Polish population aged 15–65 years [14]. Research has additionally shown that the KomPAN^®^ questionnaire may be endorsed to be used in data-driven DPs and diet quality scores to describe ordinary diets [15].

### 2.2. Demographic and Socioeconomic Data

The demographic information of the respondents comprised their gender, age, location of residence (village, town with fewer than 20,000 residents, town with 20,000–100,000 residents, or city with over 100,000 residents), and educational attainment (vocational school, secondary school, or higher education). To better describe a participant’s economic situation, two closed-ended questions were used as follows: self-assessed economic situation (below average, average, or above average) and economic situation of the household (enough money for everything we need, we live quite sparingly, or we live very frugally).

### 2.3. Diet

Considering the frequency of eating and drinking, an indicator was determined and employed to evaluate the dietary quality of patients. The Diet Quality Index (DQI) comprises 11 categories of food that may have positive impacts on health. The DQI was determined by adding the consumption frequencies, represented as daily occurrences, and subsequently transformed into a scale of 100 points.

Taking into account the eating and drinking frequency, an indicator was calculated and used to assess the quality of the diets of patients. The Diet Quality Index (DQI) consists of 11 food groups with potentially beneficial effects on one’s health (wholemeal bread, milk, fermented milk drinks, cottage cheese, fish dishes, legumes, potatoes, fruit, vegetables, canned vegetables, and fruit juices). The DQI was calculated by summing up the frequencies of consumption, expressed as the number of times per day, and was then converted into a 100-point scale. Nutritional knowledge was assessed using 25 statements regarding food and nutrition, which the participants classified as true or false [15]. When the responses were accurate, each participant received 1 point, then the total points were calculated, and each individual was categorised into one of three groups: inadequate (0–8 points); adequate (9–16 points); or excellent (17–25 points) nutritional knowledge.

The respondents also declared the number of daily eating instances (by choosing one of five answers ranging from one meal a day to five or more meals a day), eating regularly, frequency of eating between meals, and eating away from home, as well as the types of consumed alcoholic drinks and the foods consumed most often between meals.

### 2.4. Lifestyle

The subsequent aspects of a participant’s lifestyle were evaluated: smoking, exercise, and sleep duration. The past and current smoking had a binary selection of responses: yes or no. The participants selected one of three classifications to indicate their physical activity level: low—over 70% of time inactive; moderate—approximately 50% of time inactive and 50% active; and high—roughly 70% of time active or engaged in high-intensity physical work. The participants similarly selected one of six categories: <2 h/day; 2 to <4 h/day; 4 to <6 h/day; 6 to <8 h/day; 8 to <10 h/day; and ≥10 h/day. The reported sleep duration was categorised as follows: ≤6 h/day; 7–8 h/day; and ≥9 h/day. The self-reported health condition relative to individuals of similar age was evaluated using these responses: worse than peers; the same as peers; and better than peers.

### 2.5. Body Composition and Anthropometric Measurement Assessment

The International Federation for the Surgery of Obesity and Metabolic Disorders Guidelines were employed to distinguish between patients with and without a surgical indication [16]. Only individuals with a BMI ≥ 35 kg/m^2^ and associated comorbidities linked to high weight or those with a BMI ≥ 40 kg/m^2^ qualified for surgery. The participants in this study did not encompass patients who had contraindications for surgery [17]. Just before the body composition assessment, participants were prohibited from consuming a large amount of fluids, drinking alcohol for 24 h before the test, or eating foods high in sugar, salt, or fat for 12 h before the test.

Body height (cm) was assessed with a professional anthropometer while standing, ensuring the shoulders were aligned normally and the head was positioned in the horizontal Frankfurt plane [18,19]. Waist circumference (WC, cm) at the level of the umbilicus and hip circumference (HC, cm) at the widest point was measured with a tape measure. The body mass index (BMI, kg/m^2^) was determined by dividing weight (kg) by the square of height (m^2^). The subsequent parameters were directly measured through bioimpedance analysis to evaluate body composition (TANITA MS-780, Poznań, Poland): fat mass (BFM, kg; and BF, %); visceral fat level (VFL, ranges: 1–59); and body muscle mass (BMM, kg).

The degree of obesity was classified based on the BMI in both women and men (<39.9 kg/m^2^; 40–49.9 kg/m^2^; ≥50 kg/m^2^) and VFL (Medium < 13; High 13–18; Very high > 18). Due to differences in the BF and BMM between men and women, the indices were analysed in tercile groups separately for men (M) and women (W); for the BF: 1st tercile: M: <37.6%, W: <42.5%; 2nd tercile M: 37.6–42.6%, W: 42.5–47.7%; 3rd tercile M: ≥42.6%, W: ≥47.7%); and for the BMM: (1st tercile M: <77.8 kg, W: <57.4 kg; 2nd tercile M: 77.8–85.3 kg, W: 57.4–63.3 kg; 3rd tercile M: ≥85.3 kg; W: ≥63.3 kg).

### 2.6. Statistical Analysis

The Kolmogorov–Smirnov test assessed the distribution of variables, confirming it was normal. Information on continuous characteristics was presented as the mean ± standard deviation (x ± SD), while categorical characteristics were shown as percentages and counts. The chi-squared test was utilised to assess notable differences in categorical variables among the dietary patterns, while a one-way analysis of variance (ANOVA) was employed to examine significant mean differences in continuous variables across men and women.

The dietary patterns (DPs) were obtained using a principal component analysis (PCA) with a normalised Varimax value. The Kaiser–Mayer–Olkin test (KMO), which yields a KMO value of 0.7, and the Bartlett test, which achieved statistical significance (*p* < 0.0001), indicated that the analysis and number of factors were selected correctly. The PCA encompassed 24 diet-related variables. The number of DPs was identified based on the following criteria: (1) eigenvalues of the variable correlations > 1.0; (2) plot of eigenvalues; and (3) magnitude of the explained variance. The rotated factor loadings with an absolute value > |0.40| were considered pattern-specific and were used to label the patterns accordingly. The higher the values of factor loadings, the stronger the association between the participants’ diet and the DP. The percentage of adiposity and the BF and BMM terciles were analysed across the tertiles of the DPs using Pearson’s chi-squared test.

A multivariate regression verified the associations between the DPs and both the degree of obesity and body composition. The odds ratios (ORs) and 95% confidence intervals (95%CIs) were calculated. The model was created with an adjustment for potential confounders, i.e., age (a continuous variable) and gender, smoking, economic situation, and level of education (categorical variables). The modelled variables were classified based on the BMI (ref.: Degree I obesity) and VFL (ref.: Medium < 13), terciles for the BF (ref.: 1st tercile), and terciles for the BMM (ref.: 1st tercile). With respect to the participants’ adherence to the DPs, the modelled categories were T2 or T3 adherence, while the reference category (OR = 1.00) was a low adherence (i.e., T1). The statistical analysis was performed using the STATISTICA 13.1 software (StatSoft, TIBCO Gold Partner 2020, Kraków, Poland). A *p*-value < 0.05 was considered to be statistically significant.

## 3. Results

The mean age of the participants was 41.4 ± 10.1 years, for the men 43.0 ± 9.9 years, and for the women 40.9 ± 10.2 years, respectively. Three-fourths of the participants (75.2%) were women, and almost one-third lived in villages (29.7%) (Table 1). More than 70% described their financial situation as average, while more than 50% declared their household situation as ‘we live quite sparingly’. Most women (52.9%) had achieved a higher level of education. More than 75% declared their health to be worse compared to people of the same age due to the disease. More than 60% declared a low level of physical activity. Only 15.4% of the patients had never smoked. The percentage of current and former smokers did not differ significantly between men and women. Almost 60% of the participants reported sleeping 7–8 h per day.

According to the qualification of patients for BS, both the men and women showed obesity. Mean body mass was significantly higher in men than in women, whereas the body fat percentage was significantly higher in women than in men (53% vs. 40.9%, respectively; Table 2). Waist circumference was significantly higher in men than in women, whereas the hip circumference did not differ significantly between the two groups, with a mean value of about 130 cm.

Most of the participants ate four or five meals per day, but not regularly (Table 3). All the participants ate between meals, and one in every three women did so several times per day. The preferred alcoholic drink among women was wine. More than 75% of the participants declared fruit as the type of food they ate the most often between meals.

The PCA identified three primary DPs, which explained a total of 37% of the variance in eating habits (Table 4). The first DP was labelled as ‘Sandwiches & Sweets’. It showed a positive correlation with the consumption of cold cuts and sausages, white and refined bread, and sweets. The second DP, labelled ‘Fast Food, Convenience Food & Alcohol’, was associated primarily with the consumption of fast foods, canned meats, alcohol, lard, sweetened drinks, cheese, and fried foods. The third DP, labelled ‘prudent’, correlated with the consumption of white meats, vegetables, fruit, and wholemeal groats.

An analysis of the Diet Quality Index (DQI) indicated significant differences between the tercile groups of the Sandwiches & Sweets DP (Table 5). Specifically, the participants in T3 displayed low levels of healthy traits in the DQI significantly more frequently than those in T1 and T2. No participant showed a high intensity of healthy traits among all of the analysed patterns. Insufficient nutritional knowledge was observed among almost 20% of the participants across all DPs. The use of the Prudent DP was associated with significant differences in nutritional knowledge. The percentage of participants with insufficient nutritional knowledge in T3 was significantly higher than in T2. Conversely, the participants in T2 of the Prudent DP had sufficient knowledge significantly more often than those in T1.

An analysis of the Sandwiches & Sweets DP only found significant differences in the levels of VFL, where the percentage of participants with a very high VFL was 1.5 times higher in T2 than in T1 and T3 (Table 6). The Fast Food, Convenience Food & Alcohol DP only displayed significant differences according to the degree of obesity: the percentage of patients with Degree III obesity was three times higher in T1 than in T2. Following the Prudent DP differentiated the participants’ degree of obesity and body composition the most. The percentage of patients with Degree II obesity was significantly higher in T1 than in T2 and T3, whereas there were significantly more patients with Degree III obesity in T3 than in T1 and T2. Patients with the higher body fat percentage (T3 of BF%) followed the Prudent DP significantly more frequently in T3 than in T2. In turn, the percentage of patients in the T2 of BMM was significantly lower in T3 than in T1 and T2.

The results of multivariate regression analysis showed that confounding factors, smoking, gender, age, economic situation, and level of education significantly influenced the relationships between adherence to the Prudent Dietary Pattern (DP) and both the degree of obesity and body composition (Table 7). The analysis of adjusted odds ratios revealed that adherence to the Prudent DP was associated with a decreased likelihood of Degree II obesity (specifically, adjusted odds ratios of 0.20 with a 95% CI of 0.06–0.70 in T2 and 0.20 with a 95% CI of 0.05–0.86 in T3) as well as elevated body muscle mass (BMM) in the second tertile (adjusted odds ratio of 0.17 with a 95% CI of 0.03–0.92 in T3). Additionally, the analysis of adjusted odds ratios indicated specific modifying effects attributed to both the economic situation and the level of education (see Supplementary Table S1).

## 4. Discussion

According to our knowledge, this is the first study to attempt to identify DPs in patients who have qualified for a bariatric procedure. Significant differences were found between the DPs and the degree of obesity as well as between the DPs and body composition. The Prudent DP identified in this study primarily involved a high consumption of healthy products: meats, vegetables, fruit, and wholemeal groats. Following the Prudent DP, the degree of obesity and body composition was differentiated the most. The highest percentage of patients with Degree III obesity and the highest FM followed the most beneficial DP (T3 of the Prudent DP). Following this diet before BS may contribute to a reduction in visceral fat and the maintenance of lean body mass [20]. A major part of the evidence suggests that even an insignificant weight loss unrelated to the disease has a beneficial effect and may lead to an improvement in the patient’s metabolic health and public health and a reduction in the costs of healthcare [21,22]. Patients with a higher degree of obesity were able to follow a more beneficial DP due to both their higher awareness of the risks related to morbidity in obesity and their fear of exclusion from the procedure in the case of insufficient weight loss prior to surgery. Some studies have confirmed that the percentage of patients who feel a need to lose weight differs depending on the BMI category, i.e., it increases along with the BMI [23].

The lack of a correlation between a more beneficial DP and body composition (e.g., a lower fat mass) seems surprising because the patients with Degree III obesity constituted the significantly most numerous group in the T3 of the Prudent DP. Such healthy eating behaviour may be related to the amount of weight that a surgeon instructs the patients to lose before the procedure. Furthermore, about 90% of the patients showed a low intensity of traits from the Healthy Diet Index. Research indicates that the most common methods of losing weight directly before a bariatric procedure are low-energy diets, which usually involve an intake of 800–1200 and 500–800 kcal/day [20,24]. However, there is insufficient data concerning the effectiveness of this DP in patients prior to BS, whose diets are usually improperly balanced and who do not change their eating behaviour after the procedure [25].

The Fast Food, Convenience Food & Alcohol DP was characterised by high consumption of fast foods, canned meats, alcohol, lard, sweetened drinks, cheese, and fried foods. It was also associated with a higher likelihood of Degree III obesity and a very high visceral fat level. This DP usually results in an excessive amount of fat in the diet, especially saturated fatty acids (SFAs). SFAs may affect an individual’s gene expression, lead to dysbiosis of the gut microbiota, cause systemic inflammation, and contribute to the onset of metabolic disorders and chronic diseases [26]. Studies conducted by other researchers indicate that most patients perceive a high-fat diet as tasty, and as a result, they eat more in order to feel sated [27]. In addition, the energy cost of depositing fats in the fat tissue is low. Clinical studies have confirmed the harmful effects of long-term consumption of high-fat, low-fibre meals and the resulting tendency to develop hyperglycaemia, increased insulinemia, increased LDL, and decreased HDL [28,29]. In a study by Li et al. [30], four strains of experimental mice treated with HFD were used to explore the impact of mouse strain on the lipid profile, glucose level, and major inflammatory cytokines. It was found that HFD-fed Kunming and ICR mice obtained a significantly higher body weight than the controls. Alcohol was strongly correlated with the Fast Food, Convenience Food & Alcohol DP and was shown to potentially affect the intake of energy by inhibiting the actions of leptin and glucagon-like peptide 1, which are responsible for the feeling of satiety [31].

Similar relationships were observed for the Sandwiches & Sweets DP, which included the most patients with a very high VFL. A chronic positive energy balance, especially one caused by highly processed products, leads to adipocyte stress [32]. The inability of the fat tissue to uptake lipids from a person’s diet results in their excessive transfer to other tissues after meals [33]. Consequently, an increased concentration of fatty acids in the blood stimulates their uptake in the liver, which leads to the formation of triglycerides and the development of dyslipidaemia. Research has also shown that a regular intake of sweets and other products containing glucose–fructose syrup increases one’s appetite [34].

A dietary error that impacts the body’s energy balance is eating between meals, which contributes to overconsumption [35]. Frequent consumption of meals (4–5 times per day) was shown to help prevent weight gain, whereas eating snacks (sweets, salty sticks, and nuts) between main meals may negatively affect the energy balance. Eating between meals has no physiological foundation (it is not caused by hunger), but rather, it results from a habit, craving, stress, or the smell of food. In a different article published by the authors of this study, 68% of the participants ate the recommended number of meals, but almost 60% of the men and women did not eat regularly. Similar results were obtained in a pilot study conducted by the same team [36]. Furthermore, almost half of the participants were observed to eat between meals one or several times per day, usually fruit or sweet and salty snacks, which likely translated into difficulties with controlling their energy intake. A study found that with free access to food (ad libitum), the daily intake of calories may increase by as much as 20% [35]. However, Vatanparast et al. [37] did not observe any significant differences in obesity measures (i.e., the BMI and the BMI z-scores) between snack consumers and non-consumers in both children and adults from Canada. Another study found that the correlations were related to the time of snack consumption. Frequent eating between meals in the evening was associated with an increased BMI [38], which suggested that eating between meals in the evening was an obesogenic behaviour. The results of our research verified that socioeconomic factors may influence the relationships between Prudent DP and obesity degree as well as body composition, partially aligning with findings from other researchers [1]. Foods of lower nutritional value and lower-quality diets generally cost less and are more frequently selected by individuals from lower socioeconomic backgrounds, whereas maintaining a healthier diet has consistently been linked to increased expenses [39].

The primary limitation of this study was a lack of monitoring of the energy balance in the diets of the patients who had qualified for a bariatric procedure. Dietary errors should be monitored both quantitatively and qualitatively to allow for a more detailed characterisation of the onset mechanisms of obesity. Another limitation of the work was the lack of detailed analysis of psychological features (depression and anxiety). Obesity is commonly considered a psychological problem. We therefore assumed that all our patients may have such problems [40,41].

## 5. Conclusions

The results of this study demonstrated that patients who qualified for a bariatric procedure followed different diets, and different DPs could be identified.

In this group, DPs were associated with the degree of obesity and body composition. Patients with a higher degree of obesity followed a more beneficial DP, which was likely due to their higher awareness of the risks of morbidity in obesity and of post-surgical complications. Factors such as economic situation and level of education may modulate the relationship between certain DPs and the degree of obesity and body composition in bariatric surgery candidates.

Providing personalised nutritional counselling to BS candidates by helping them to make correct dietary decisions may improve both the energy and nutritional value of their diets.

## Figures and Tables

**Table 1:** Characteristics of the participants (%).

Characteristics		Total *n* = 117	Men *n* = 29	Women *n* = 88	*p*-Value
Age [years]	x ± SD	41.4 ± 10.1	43.0 ± 9.9	40.9 ± 10.2	ns **
Place of residence					
	Village	29.7	24.1	35.2	ns
	Town < 20,000 residents	28.5	31.0	26.1	
	Town 20,000–100,000 residents	14.4	17.3	11.4	
	City > 100,000 residents	27.4	27.6	27.3	
Education					
	Vocational	36.9	38.5	35.3	<0.05
	Secondary	23.2	34.6	11.8	
	Higher	39.9	26.9	52.9	
Economic situation of the household *					
	Enough money for everything we need	25.2	27.6	22.7	ns
	We live quite sparingly	54.8	51.7	58.0	
	We live very frugally	20.0	20.7	19.3	
Self-assessed economic situation					
	Below average	6.7	6.9	4.5	ns
	Average	71.4	62.1	80.7	
	Above average	22.8	31.0	14.8	
Self-assessed health status compared to people of the same age					
	Worse than peers	75.5	82.8	68.2	ns
	The same	23.3	17.2	29.5	
	Better than peers	1.2	0.0	2.3	
Self-assessed physical activity					
	Low	62.3	62.1	62.5	ns
	Moderate	29.1	34.4	23.9	
	High	8.6	3.5	13.6	
Hours of sleep on weekdays					
	Less than 6 h/day	38.3	37.9	38.6	ns
	From 7 to 8 h/day	57.2	58.6	55.7	
	More than 9 h/day	4.5	3.5	5.7	
Hours of sleep on weekends					
	Less than 6 h/day	16,5	17.2	15.9	ns
	From 7 to 8 h/day	66.3	69.0	63.6	
	More than 9 h/day	17.1	13.8	20.5	
Currently smoking		22.3	24.1	20.5	ns
Smoking in the past		62.3	65.5	59.1	ns

* According to the Polish Central Statistical Office (GUS), www.stat.gov.pl (accessed on 10 September 2024); ns—statistically non-significant difference; Pearson’s chi-squared test was used to verify differences in the sample; x—mean; SD—standard deviation; ** *p*-value—ANOVA test was used.

**Table 2:** Bioelectrical impedance analysis and anthropometrics parameters according to sex.

Variable	Total *n* = 117	Men *n* = 29	Women *n* = 88	*p*-Value
	x ± SD	x ± SD	x ± SD	
Weight [kg]	125.4 ± 24.2	150.0 ± 24.2	117.3 ± 17.9	<0.001
Height [cm]	168.9 ± 8.6	179.0 ± 7.4	165.5 ± 6.0	<0.001
WC [cm]	131.9 ± 15.9	144.5 ± 13.6	127.4 ± 14.3	<0.001
HC [cm]	130.3 ± 13.7	132.5 ± 14.1	129.5 ± 13.6	ns
BMI [kg/m^2^]	43.8 ± 6.8	46.9 ± 7.4	42.8 ± 6.3	<0.01
BF [%]	43.5 ± 6.5	40.9 ± 5.0	53.0 ± 6.7	<0.05
BFM [kg]	55.3 ± 15.0	61.4 ± 17.0	57.0 ± 13.7	<0.05
BMM [kg]	67.4 ± 12.5	82.4 ± 8.1	61.9 ± 8.8	<0.001
VFL	17.4 ± 7.2	26.0 ± 6.4	14.2 ± 4.2	<0.001

BF%—body fat percentage; BFM—body fat mass; BMI—body mass index; BMM—body muscle mass; HC—hip circumference; VFL—visceral fat level; WC—waist circumference; ns—statistically non-significant difference; SD—standard deviation; *p*-value—ANOVA test was used; x—mean.

**Table 3:** Overview of selected eating behaviours in the studied men and women.

Characteristics		Total *n* = 117	Men *n* = 29	Women *n* = 88	*p*-Value
Number of meals consumed per day					
	1	1.7	3.5	0.0	ns
	2	5.7	3.5	7.9	
	3	24.6	24.1	25.0	
	4	36.0	44.8	27.3	
	5 or more	32.0	24.1	39.8	
Eating regularly					
	No	58.5	62.0	55.7	ns
	Yes, but only some meals	35.6	34.5	36.4	
	Yes, all meals	5.9	3.5	7.9	
Frequency of eating between meals					
	Never	0.0	0.0	0.0	<0.05
	1–3 times per month	9.8	17.2	2.3	
	Once per week	9.2	3.5	14.8	
	Several times per week	34.3	34.5	34.1	
	Once per week	23.4	27.6	19.3	
	Several times per day	23.4	17.2	29.5	
Eating away from home					
	Never	25.6	31.0	23.9	ns
	1–3 times per month	46.2	37.9	48.9	
	Once per week	16.2	13.8	17.0	
	Several times per week	7.7	10.3	6.8	
	Once per day	2.6	0.0	3.4	
	Several times per day	1.7	6.9	0.0	
Types of alcoholic drinks consumed					
	Beer	15.0	21.7	8.3	<0.05
	Wine	36.7	21.7	51.7	
	Drinks	23.5	30.4	16.7	
	Strong alcohol	24.8	26.2	23.3	
Type of food eaten between meals					
Fruits		76.6	82.8	70.4	ns
Sweet snacks		51.4	44.8	57.9	ns
Salty snacks		48.6	48.3	48.9	ns
Nuts, almonds, seeds, pips		45.7	44.8	46.6	ns
Vegetables		32.6	41.4	23.9	ns
Unsweetened dairy drinks and desserts		19.5	20.7	18.2	ns

ns—statistically non-significant difference; Pearson’s chi-squared test was used to verify differences in the sample.

**Table 4:** Factor-loading matrix for major dietary patterns.

	Dietary Patterns (DPs)		
	Factor I	Factor II	Factor III
	Sandwiches & Sweets DP	Fast Food, Convenience Food & Alcohol DP	Prudent DP
Cold cuts and sausages	**0.54**	0.27	0.15
Sweets	**0.52**	0.16	−0.29
Refined bread	**0.50**	0.37	−0.16
Fast food	0.00	**0.74**	0.17
Canned meats	−0.25	**0.69**	−0.13
Alcohol	0.09	**0.57**	0.12
Lard	−0.16	**0.53**	−0.19
Sweetened drinks	0.19	**0.49**	−0.24
Cheese	0.24	**0.47**	−0.34
Fried foods	0.29	**0.45**	−0.39
Legumes	**−0.51**	0.40	−0.33
Red meats	0.17	0.39	−0.20
Fish	**−0.66**	0.11	−0.20
Wholemeal groats, flakes, and pasta	**−0.56**	−0.09	**0.41**
White meats	0.00	0.06	**0.64**
Vegetables	−0.07	−0.12	**0.61**
Fruit	0.06	0.08	**0.60**
Energy drinks	−0.06	0.19	**−0.53**
Butter	0.01	0.24	**−0.47**
Fermented milk beverages	−0.18	**−0.46**	0.22
Cottage cheese	0.17	**−0.64**	0.03
Wholegrain bread	−0.33	**−0.53**	0.20
Refined groats, rice, and pasta	0.22	0.08	0.06
Milk	−0.14	0.15	0.03
Percentage of variance explained (%)	13	13	11

Bold—The rotated factor loadings with an absolute value > |0.40| were considered pattern-specific and were used to label the patterns accordingly.

**Table 5:** Diet Quality Index (DQI) and nutritional knowledge (%) by adherence to the dietary patterns (DPs) in the study sample (*n* = 117).

Variable	Total *n* = 117	Factor I Sandwiches & Sweets DP			*p*-Value	Factor II Fast Food, Convenience Food & Alcohol DP			*p*-Value	Factor III Prudent DP			*p*-Value
		T1 *n* = 39	T2 *n* = 40	T3 *n* = 38		T1 *n* = 39	T2 *n* = 38	T3 *n* = 40		T1 *n* = 39	T2 *n* = 40	T3 *n* = 38	
DQI													
Low levels of healthy traits	88.0	71.8 ^a,b^	92.5 ^a^	100.0 ^b^	<0.001	84.6	86.8	92.5	ns	92.3	86.8	85.0	ns
Low intensity of unhealthy features and healthy features	12.0	28.2 ^a,b^	7.5 ^a^	0.0 ^b^		15.4	13.2	7.5		7.7	13.2	15.0	
High intensity of healthy features	0.0	0.0	0.0	0.0		0.0	0.0	0.0		0.0	0.0	0.0	
Nutritional knowledge level													
Insufficient	19.7	18.0	25.0	15.8	ns	17.9	10.5 ^c^	30.0 ^c^	ns	20.5	7.9 ^c^	30.0 ^c^	<0.05
Satisfactory	64.1	69.2	60.0	63.2		64.1	68.4	60.0		69.2	65.8	57.5	
Good	16.2	12.8	15.0	21.0		17.9	21.1	10.0		10.3 ^a^	26.3 ^a^	12.5	

ns—statistically non-significant difference; Pearson’s chi-squared test was used to verify differences in sample distribution across the levels of adherence to DP; T—tertile of the DPs in the study sample; ^a^—a statistically significant difference was found between T1 and T2; ^b^—a statistically significant difference was found between T1 and T3; ^c^—a statistically significant difference was found between T2 and T3.

**Table 6:** Degree of obesity and body composition by adherence to the dietary patterns (DPs) in the study sample (*n* = 117).

Variable	Total *n* = 117	Factor I Sandwiches & Sweets DP			*p*-Value	Factor II Fast Food, Convenience Food & Alcohol DP			*p*-Value	Factor III Prudent DP			*p*-Value
		T1 *n* = 39	T2 *n* = 40	T3 *n* = 38		T1 *n* = 39	T2 *n* = 38	T3 *n* = 40		T1 *n* = 39	T2 *n* = 40	T3 *n* = 38	
BMI													
Degree I obesity (<39.9 kg/m^2^)	27.4	35.9	20.0	26.3	ns	30.8	23.7	27.5	<0.05	17.9 ^a^	39.5 ^a^	25.0	<0.01
Degree II obesity (40–49.9 kg/m^2^)	56.4	51.3	60.0	57.9		43.6 ^a^	68.4 ^a^	57.5		76.9 ^a,b^	47.3 ^a^	45.0 ^b^	
Degree III obesity (≥50 kg/m^2^)	16.2	12.8	20.0	15.8		25.6 ^a^	7.9 ^a^	15.0		5.2 ^b^	13.2 ^c^	30.0 ^b,c^	
VFL													
Medium (<13)	25.0	28.6	14.3 ^c^	33.3 ^c^	<0.05	21.9	26.7	26.5	ns	22.6	27.3	25.0	ns
High (13–18)	36.5	39.3	34.3	36.4		28.1 ^a^	46.7 ^a^	35.3		48.4 ^b^	33.3	28.1 ^b^	
Very high (>18)	38.5	32.1 ^a^	51.4 ^a,c^	30.3 ^c^		50.0 ^a^	26.7 ^a^	38.2		29.0	39.4	46.9	
BF													
1st tercile (M: <37.6%; W: <42.5%)	32.3	39.3	28.6	30.3	ns	34.4	33.3	29.4	ns	38.7	30.3	28.1	<0.05
2nd tercile (M: 37.6–42.6%; W: 42.5–47.7%)	33.3	21.4 ^b^	34.3	42.4 ^b^		28.1	33.3	38.2		25.8 ^a^	45.5 ^a^	28.1	
3rd tercile (M: ≥42.6%; W: ≥47.7%)	34.4	39.3	37.1	27.3		37.5	33.3	32.4		35.5	24.2 ^b^	43.8 ^b^	
BMM													
1st tercile (M: <77.8 kg; W: <57.4 kg)	32.3	21.4	37.1	36.3	ns	37.5	26.7	32.3	ns	29.0	21.2 ^c^	46.9 ^c^	<0.05
2nd tercile (M: 77.8–85.3 kg; W: 57.4–63.3 kg)	33.3	42.9	31.4	27.3		25.0	40.0	25.3		45.2 ^b^	36.4 ^c^	18.7 ^b,c^	
3rd tercile (M: ≥85.3 kg; W: ≥63.3 kg)	34.4	35.7	36.5	36.4		37.5	33.3	35.4		25.8	42.4	34.4	

BF—body fat percentage; BMI—body mass index; BMM—body muscle mass; VFL—visceral fat level; ns—statistically non-significant difference; Pearson’s chi-squared test was used to verify differences in the sample distribution across the levels of adherence to DP; T—tercile of the DPs in the study sample; ^a^—a statistically significant difference was found between T1 and T2; ^b^—a statistically significant difference was found between T1 and T3; ^c^—a statistically significant difference was found between T2 and T3.

**Table 7:** Adjusted ^1^ associations between dietary patterns (DPs) and degree of obesity and body composition (*n* = 117): odds ratios (95% confidence interval).

Dietary Paterns ^2^		BMI			VFL			BF			BMM		
		Degree I Obesity	Degree II Obesity	Degree III Obesity	Medium	High	Very High	1st Tercile	2nd Tercile	3rd Tercile	1st Tercile	2nd Tercile	3rd Tercile
Sandwiches & Sweets	T1	ref.	ref.	ref.	ref.	ref.	ref.	ref.	ref.	ref.	ref.	ref.	ref.
	T2	ref.	0.56 (0.18–1.77)	1.87 (0.34–9.86)	ref.	1.27 (0.25–6.56)	6.99 (0.82–59.25)	ref.	1.86 (0.47–7.43)	1.39 (0.37–5.20)	ref.	0.35 (0.09–1.37)	0.37 (0.09–1.56)
	T3	ref.	0.63 (0.21–1.91)	1.61 (0.29–9.07)	ref.	2.35 (0.55–10.09)	3.99 (0.47–33.98)	ref.	2.66 (0.66–10.71)	0.84 (0.21–3.31)	ref.	0.29 (0.07–1.20)	0.52 (0.12–2.17)
Fast Food, Convenience Food & Alcohol	T1	ref.	ref.	ref.	ref.	ref.	ref.	ref.	ref.	ref.	ref.	ref.	ref.
	T2	ref.	3.18 (0.89–11.3)	0.20 (0.01–3.13)	ref.	1.15 (0.22–6.11)	0.29 (0.05–1.59)	ref.	1.21 (0.29–4.92)	0.95 (0.25–3.67)	ref.	3.7 (0.82–16-72)	1.11 (0.27–4.56)
	T3	ref.	1.78 (0.52–6.06)	1.25 (0.23–6.82)	ref.	0.98 (0.21–4.65)	0.28 (0.05–1.58)	ref.	1.63 (0.43–6.22)	1.37 (0.35–5.42)	ref.	2.13 (0.49–9.14)	0.66 (0.16–2.65)
Prudent	T1	ref.	ref.	ref.	ref.	ref.	ref.	ref.	ref.	ref.	ref.	ref.	ref.
	T2	ref.	0.20 * (0.06–0.71)	1.17 (0.18–7.56)	ref.	0.29 (0.06–1.39)	6.99 (0.82–59.25)	ref.	2.17 (0.56–8.45)	1.01 (0.26–3.95)	ref.	0.62 (0.14–2.73)	3.46 (0.78–15.37)
	T3	ref.	0.20 * (0.05–0.86)	4.20 (0.71–24.9)	ref.	0.17 (0.03–1.16)	3.99 (0.47–33.98)	ref.	1.73 (0.35–8.54)	1.76 (0.40–7.68)	ref.	0.17 * (0.03–0.92)	0.89 (0.19–4.19)

^1^ Odds ratios adjusted for age (a continuous variable) and gender, smoking, place of residence, economic situation, and education (categorical variables). ^2^ Dietary patterns are based on subjects’ tertiles. Statistical significance (Wald test): * *p* < 0.05.

## Data Availability

Data presented in this study are available on request from the corresponding author.

## Supplementary Materials

The following supporting information can be downloaded at: https://www.mdpi.com/article/10.3390/nu17040716/s1, Table S1. Adjusted ^1^ associations between dietary patterns (DPs) and degree of obesity and body composition (*n* = 117): odds ratios (95% Confidence Interval).

## Abbreviations

The following abbreviations are used in this manuscript:

MDPI	Multidisciplinary Digital Publishing Institute
DOAJ	Directory of Open Access Journals
TLA	Three Letter Acronym
LD	Linear Dichroism
