# Supplementary material for: Dietary Patterns and Nutritional Status in Bariatric Surgery Candidates—A Cross-Sectional Study
Source: Nutrients. 2025 Feb 18;17(4):716. doi: 10.3390/nu17040716 (PMC11858383; doi:10.3390/nu17040716)
Supplement: Supplementary file 1 [file nutrients-17-00716-s001.zip › nutrients-3469281-supplementary.pdf]

Supplementary Table S1. Adjusted[1] associations between dietary patterns (DPs) and degree of obesity and body composition (n = 117): odds ratios (95% Confidence Interval).

Dietary paterns[2]		BMI			VFL			BF			BMM		
		Degree I obesity	Degree II obesity	Degree III obesity	Medium	High	Very high	1st tercile	2nd tercile	3rd tercile	1st tercile	2nd tercile	3rd tercile
**Sandwiches & sweets**	T1	ref.	ref.	ref.	ref.	ref.	ref.	ref.	ref.	ref.	ref.	ref.	ref.
	T2	ref.	0,56 (0.18-1.77)	1.87 (0.34-9,86)	ref.	1.27 (0.25-6.56)	6.99 (0.82-59.25)	ref.	1.86 (0.47-7.43)	1.39 (0.37-5.20)	ref.	0.35 (0.09-1.37)	0.37 (0.09-1.56)
	T3	ref.	0,63 (0.21-1.91)	1.61 (0.29-9.07)	ref.	2.35 (0.55-10.09)	3.99 (0.47-33.98)	ref.	2.66 (0.66-10.71)	0.84 (0.21-3.31)	ref.	0.29 (0.07-1.20)	0.52 (0.12-2.17)
Smoking		ref.	1.38 (0.38-4.99)	2.05 (0.39-10.83)	ref.	1.80 (0.33-9.89)	0.88 (0.27-6.09)	ref.	0.98 (0.22-4.46)	1.94 (0.49-7.63)	ref.	0.72 (0.16-3.29)	0.46 (0.12-1.85)
Gender		ref.	1.34 (0.33-5.39)	0,15* (0.02-0.87)	ref.	-	-	ref.	0.70 (0.17-2.96)	0.65 (0.16-2.59)	ref.	1.03 (0.23-4.61)	0.89 (0.21-3.87)
Age		ref.	0.99 (0.95-1.05)	0.97 (0.89-1.04)	ref.	0.99 (0.93-1.07)	1.11* (1.03-1.21)	ref.	1.00 (0.95-1.06)	1.01 (0.96-1.07)	ref.	0.98 (0.93-1.04)	0.94* (0.89-0.99)
Average economic situation		ref.	0.97* (0.01-0.89)	1.38 (0.08-23.46)	ref.	0.09 (0.004-2.02)	3.91 (0.22-68.09)	ref.	1.34 (0.15-11.97)	1.77 (0.12-26.09)	ref.	3.08 (0.21-44.83)	3.88 (0.32-47.46)
Above average economic situation		ref.	0.10 (0.01-1.39)	1.07 (0.04-28.91)	ref.	0.13 (0.004-4.19)	27.79 (0.89-870.08)	ref.	0.67 (0.05-9.28)	0.66 (0.31-14.18)	ref.	1.39 (0.06-33.7)	4.16 (0.27-64.35)
Secondary education		ref.	2.13 (0.34-13.28)	1.17 (0.09-15.78)	ref.	2.43 (0.17-34.20)	0.16 (0.01-2.27)	ref.	0.66 (0.12-3.55)	1,19 (0.21-6.83)	ref.	0.69 (0.09-4.93)	0.31 (0.05-1.77)
Higher education		ref.	7.39* (1.08-50.72)	1.23 (0.09-15.78)	ref.	14.21 (0.93-216.89)	0.03** (0.002-0.43)	ref.	0.88 (0.14-5.50)	1.99 (0.30-12.96)	ref.	0.81 (0.89-7.29)	0.18 (0.03-1.17)
**Fast Food, Convenience Food & Alcohol**	T1	ref.	ref.	ref.	ref.	ref.	ref.	ref.	ref.	ref.	ref.	ref.	ref.
	T2	ref.	3.18 (0.89-11.3)	0.20 (0.01-3.13)	ref.	1,15 (0.22-6.11)	0.29 (0.05-1.59)	ref.	1.21 (0.29-4.92)	0.95 (0.25-3.67)	ref.	3.7 (0.82-16-72)	1.11 90.27-4.56)
	T3	ref.	1.78 (0.52-6.06)	1.25 (0.23-6.82)	ref.	0.98 (0.21-4.65)	0.28 (0.05-1.58)	ref.	1.63 (0.43-6.22)	1.37 (0.35-5.42)	ref.	2.13 (0.49-9.14)	0.66 (0.16-2.65)
Smoking		ref.	0.84 (0.22-3.18)	1.83 (0.29-11.64)	ref.	0.63 (0.10-3.79)	0.84 (0.15-4.89)	ref.	0.95 (0.21-4.36)	1.91 (0.49-7.44)	ref.	0.74 (0.16-3.37)	0.48 (0.12-1.99)
Gender		ref.	0.56 (0.14-2.34)	0.16 (0.02-1.06)	ref.	-	-	ref.	0.76 (0.18-3.11)	0.62 (0.16-2.45)	ref.	0.63 (0.13-3.00)	0.96 (0.22-4.13)
Age		ref.	1.00 (0.96-1.05)	0.97 (0.91-1.05)	ref.	1.00 (0.94-1.07)	1.10* (1.02-1.19)	ref.	1.01 (0.95-1.06)	1.01 (0.96-1.07)	ref.	0.99 (0.93-1.04)	0.94* 90.88-0.99)
Average economic situation		ref.	13.78* (1.38-138.07)	0.57 (0.02-14.66)	ref.	11.91 (0.66-214.64)	3.99 (0.25-64.6)	ref.	1.29 (0.14-11.87)	1.79 (0.11-28.51)	ref.	3.35 (0.24-46.54)	3.64 (0.29-45.17)
Above average economic situation		ref.	11.59 (0.79-171.14)	0.42 (0.01-15.64)	ref.	8.42 (0.32-221.45)	22.91 (0.85-619.73)	ref.	0.61 (0.04-8.72)	0.71 (0.03-14.89)	ref.	1.26 (0.06-28.49)	3.96 (0.25-65.80)
Secondary education		ref.	0.58 (0.09-3.62)	1.16 (0.09-14.85)	ref.	0.34 (0.02-5.20)	0.18 (0.01-2.66)	ref.	0.80 (0.15-4.28)	1.18 (0.20-6.93)	ref.	1.33 (0.17-10.34)	0.25 (0.04-1.62)
Higher education		ref.	0.15*	1.31	ref.	0.07	0.04*	ref.	0.87	1.83	ref.	1.46	0.18

			(0.02-0.98)	(0.10-17.04)		(0.004-1.12)	(0.003-0.51)		(0.14-5.34)	(0.29-11.44)		(0.15-13.90)	(0.03-1.24)
**Prudent**	T1	ref.	ref.	ref.	ref.	ref.	ref.	ref.	ref.	ref.	ref.	ref.	ref.
	T2	ref.	0.20* (0.06-0.71)	1.17 (0.18-7.56)	ref.	0.29 (0.06-1.39)	6.99 (0.82-59.25)	ref.	2.17 (0.56-8.45)	1.01 (0.26-3.95)	ref.	0.62 (0.14-2.73)	3.46 (0.78-15.37)
	T3	ref.	0.20* (0.05-0.86)	4.20 (0.71-24.9)	ref.	0.17 (0.03-1.16)	3.99 (0.47-33.98)	ref.	1.73 (0.35-8.54)	1.76 (0.40-7.68)	ref.	0.17* (0.03-0.92)	0.89 (0.19-4.19)
Smoking		ref.	1.36 (0.03-56.7)	0.69 (0.11-4.40)	ref.	1.23 (0.16-9.43)	0.88 (0.13-6.09)	ref.	0.73 (0.13-4.01)	1.53 (0.30-5.99)	ref.	1.93 (0.31-4.92)	0.86 (0.17-4.23)
Gender		ref.	0.54 (0.13-2.32)	0.19* (0.05-0.80)	ref.	-	-	ref.	0.83 (0.21-3.12)	0.77 (0.19-3.17)	ref.	0.79 (0.18-3.60)	0.99 (0.23-4.22)
Age		ref.	1.00 (0.95-1.06)	0.97 (0.90-1.05)	ref.	1.00 (0.94-1.08)	1.14* (1.03-1.21)	ref.	1.01 (0.95-1.07)	1.01 (0.96-1.06)	ref.	0.99 (0.3-1.04)	0.94* (0.88-0.99)
Average economic situation		ref.	23.22** (2.05-262.85)	0.89 (0.03-23.71)	ref.	25.23* (1.07-595.24)	3.92 (0.22-68.09)	ref.	0.97 (0.10-9.15)	1.73 (0.13-24.05)	ref.	4.21 (0.30-59.23)	2.93 (0.24-36.57)
Above average economic situation		ref.	17.88* (1.11-287.86)	1.39 (0.03-57.22)	ref.	12,45 (0.41-375.33)	27.80 (0.89-870.08)	ref.	0.60 (0.04-8.43)	0.66 (0.03-12.84)	ref.	1.86 (0.08-41.3)	3.43 (0.22-54.63)
Secondary education		ref.	0.54 (0.09-3.35)	1.79 (0.09-32.46)	ref.	0.35 (0.02-5.13)	0.16 (0.01-2.27)	ref.	0.63 (0.12-3.45)	1.08 (0.19-6.21)	ref.	1.02 (0.13-7.92)	0.23 (0.04-1.37)
Higher education		ref.	0.17 (0.03-1.12)	1.02 (0.06-17.36)	ref.	0.05 (0.004-0.83)	0.23* (0.002-0.43)	ref.	0.68 (0.11-4.31)	1.69 (0.27-10.60)	ref.	1.12 (0.11-11.71)	0.16 (0.02-1.09)

[1]odds ratios adjusted for age (a continuous variable) and gender, smoking, economic situation and education (categorical variables); [2]Dietary Patterns are based on subjects' tertile Statistical significance (Wald test): *p <0.05, **p <0.01.
